# Prevalence of trypanosomes and selected symbionts in tsetse species of eastern Zambia

**DOI:** 10.1017/S0031182022000804

**Published:** 2022-09

**Authors:** Gloria M. Mulenga, Boniface Namangala, Bruce Gummow

**Affiliations:** 1 Department of Veterinary Services, Kakumbi Tsetse and Trypanosomiasis Research Station, Mfuwe, Zambia; 2 College of Public Health Medical and Veterinary Sciences, James Cook University, Townsville, Queensland, Australia; 3 Department of Paraclinical Studies, The University of Zambia, School of Veterinary Medicine, Lusaka, Zambia; 4 Faculty of Veterinary Science, University of Pretoria, Pretoria, South Africa

**Keywords:** prevalence, symbiont, trypanosome, tsetse, Zambia

## Abstract

Insect symbionts have attracted attention for their potential use as anti-parasitic gene products in arthropod disease vectors. While tsetse species of the Luangwa valley have been extensively studied, less is known about the prevalence of symbionts and their interactions with the trypanosome parasite. Polymerase chain reaction was used to investigate the presence of *Wolbachia* and *Sodalis* bacteria, in tsetse flies infected with trypanosomes (*Trypanosoma vivax, Trypanosoma congolense* and *Trypanosoma brucei*). Out of 278 captured tsetse flies in eastern Zambia, 95.3% (*n* = 265, 95% CI = 92.8–97.8) carried endosymbionts: *Wolbachia* (79.1%, 95% CI 73.9–83.8) and *Sodalis* (86.3%, 95% CI 81.7–90.1). Overall, trypanosome prevalence was 25.5% (*n* = 71, 95% CI = 20.4–30.7), 10.8% (*n* = 30, 95% CI 7.1–14.4) for *T. brucei,* 1.4% (*n* = 4, 95% CI = 0.4–3.6) for both *T. congolense* and *T. vivax*, and 0.7% (*n* = 2, 95% CI 0.1–2.6) for *T. b. rhodesiense.* Out of 240 tsetse flies that were infected with *Sodalis,* trypanosome infection was reported in 40 tsetse flies (16.7%, 95% CI = 12.0–21.4) while 37 (16.8%, 95% CI 11.9–21.8) of the 220 *Wolbachia* infected tsetse flies were infected with trypanosomes. There was 1.3 times likelihood of *T. brucei* infection to be present when *Wolbachia* was present and 1.7 likelihood of *T. brucei* infection when *Sodalis* was present. Overall findings suggest absence of correlation between the presence of tsetse endosymbionts and tsetse with trypanosome infection. Lastly, the presence of pathogenic trypanosomes in tsetse species examined provided insights into the risk communities face, and the importance of African trypanosomiasis in the area.

## Graphical abstract

**Figure:**
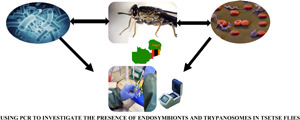

## Introduction

African trypanosomiasis, caused by protozoa belonging to the genus *Trypanosoma*, is a vector-borne disease endemic in sub-Saharan Africa. African trypanosomes are transmitted to the mammalian hosts by the bite of an infected tsetse fly (Diptera: Glossinidae) causing a fatal disease commonly known as *Nagana* in cattle and sleeping sickness in humans (WHO, 2017; Franco *et al*., 2020; Franco *et al*., 2022). *Trypanosoma congolense* is the major cause of African animal trypanosomiasis (AAT) in eastern and southern Africa whilst *Trypanosoma vivax* (together with *Trypanosoma congolense*) is a more important cause of AAT in cattle in West Africa (Cox *et al*., 2010; Laohasinnarong *et al*., 2015; Mulenga *et al*., 2021). The 2 human-infective trypanosome sub-species are *Trypanosoma brucei gambiense* (found in west and central Africa), which accounts for over 98% of reported cases of sleeping sickness, and *Trypanosoma brucei rhodesiense* (found in eastern and southern parts of Africa, including Zambia), which only accounts for less than 2% of reported cases (Nakamura *et al*., 2019; Franco *et al*., 2020).

Tsetse flies host the following 3 endogenous symbionts: *Wigglesworthia glossinidia*, *Sodalis glossinidius* and *Wolbachia* (Wamiri *et al*., 2013; Makhulu *et al*., 2021). *Wigglesworthia*, found in all tsetse flies, provides nutritional and immunological benefits to its tsetse host. In the absence of this bacteria, intrauterine larval development is stunted, and progeny aborted (Weiss and Aksoy, 2011). *Wigglesworthia*'s contracted genome, encodes an unusually high number of putative vitamin biosynthesis pathways, which support the theory that *Wigglesworthia* supplements its tsetse host with nutritious metabolites that are naturally present in low titres in vertebrate blood (Wang *et al*., 2009; Rio *et al*., 2012). *Sodalis* on the other hand can be found both intra- and extra-cellular in various tissues of tsetse flies, including midgut, body fat, milk gland, salivary glands and haemocoel (Doudoumis *et al*., 2017). *Sodalis* contains features associated with pathogenic lifestyles, including secretion systems, which function during the tsetse's juvenile developmental stages (Dennis *et al*., 2014). *Sodalis* can be cultured in cell free medium, and, unlike *Wigglesworthia*, it is usually absent in several natural tsetse populations. Lastly, *Wolbachia* is a wide-spread bacteria endosymbiont infecting approximately 70% of surveyed insects. It manipulates the reproductive biology of its host mechanisms, which include cytoplasmic incompatibility (CI), male killing, feminization and parthenogenesis (Wamiri *et al*., 2013).

Symbiotic interactions are widespread in insects (as well as animals and plants) and may provide an avenue for disease control. The use of biological methods for the control of vector-transmitted diseases is becoming popular globally (Ricci *et al*., 2012; Utarini *et al*., 2021). Symbionts influence several aspects of the tsetse's physiology, including reproduction, nutrition and vector competence. Several studies have suggested the involvement of insect microbiota in the ability of insect disease vectors to transmit pathogens (Geiger *et al*., 2007; Ricci *et al*., 2012; Weiss *et al*., 2013; Hamidou Soumana *et al*., 2014; Makhulu *et al*., 2021) thus providing hope in the potential use of symbionts to control African trypanosomiasis (Medlock *et al*., 2013). The presence of tsetse microbiota in Zambia's tsetse flies has been described in studies conducted by Mbewe *et al*. (2015) and Dennis *et al*. (2014) on wild tsetse flies. While the earlier study observed significant association between present endosymbiont and trypanosome infection, the later study found it difficult to establish if some tsetse microbiota could play a role in the susceptibility of tsetse flies to trypanosomiasis infection. Little is known about the presence of symbionts in tsetse species found along the Luangwa tsetse belt of the eastern province of Zambia and the role that tsetse endosymbionts may play in the transmission and control of trypanosomiasis. Thus, the potential use of endosymbionts in trypanosomiasis control seems attractive because trypanocide-based management of *Nagana* has proven to be costly and not sustainable. Furthermore, increasing resistance of trypanosomes to the available trypanocides has also been seen to threaten the efficacy of current control approaches. The study was therefore conducted to establish the prevalence of *Sodalis* and *Wolbachia* in tsetse species found in the eastern province of Zambia, and to determine the relationship that exists between these symbionts and trypanosomiasis infected tsetse flies.

## Materials and methods

### Study area and sample collection

Polymerase chain reaction (PCR) was used in a survey of tsetse symbionts and trypanosomes in tsetse species of eastern Zambia. Taking into consideration tsetse characteristics, Epsilon traps baited with 3-n-prophyphenol and 1-octe-3-nol released at 5 g h^−1^ from open bottles and 0.5 g h^−1^ from polythene sachets, respectively, were used for collecting tsetse flies. In areas where fly density was low, flies trapped within a moving vehicle in the trapping site was used as a supplementary method to maximize catches. Traps were deployed within, and along peripheral known tsetse affected villages (Katemo, Ncheka, Nsefu, Chilanga, Chinzombo, Malama and Chikowa) of Mambwe district in Zambia's eastern Province between the years 2019 and 2020, during the dry-hot and wet-hot seasons. Deployment of traps was determined by the availability of suitable environments to maximize tsetse catches. Each trapping site was given a unique identifier and global positioning system (GPS) coordinates recorded and maintained for cross-referencing purposes. Milking of traps was done 24 h after deployment.

### Sample preparation and storage

Tsetse samples collected were stored as whole flies in well-labelled bottles containing ethanol. Each bottle contained all tsetse samples captured from one trapping site. Tsetse flies caught from supplementary techniques (e.g., moving vehicle) were stored together with samples captured from the nearest possible trapping site. Prior to storage, identification data were recorded (date of collection, location, numbers captured, sex and species). During sample preparation, captured flies were removed from ethanol storage, blotted with tissue paper towel, and left to air dry overnight at room temperature. Unique identifiers given during sample collection were maintained.

### Laboratory analysis

Total genomic deoxyribonucleic acid (DNA) was extracted from individual flies after removing wings and legs. Manufacturer's instructions on DNA extraction kits (QIAamp^®^ DNA mini kit) were followed during the extraction process. Extracted DNA was stored in 1.5 mL tubes, labelled with unique trapping numbers related to where they were trapped. The eluted DNA was stored at 4°C for use within 12 h and at −20°C for use after 12 h.

The presence of symbionts from the extracted DNA was determined using a symbiont species-specific PCR amplification assay as described by Pais *et al*. (2008). Four nanograms of the extracted DNA template was used for each PCR. For identification of *Sodalis,* HemF (ATGGGAAACAAACCATTAGCCA) and HemR (TCAAGTGACAAACAGATAAATC) primers (Pais *et al*., 2008) were used to amplify the 650-bp fragment of the haemolysin gene (accession no. AP008232). The presence of *Wolbachia* was detected by the amplification of a 610-bp fragment of the wsp gene with primers 81F (TGGTCCAATAAGTGATGAAGAAAC) and 691R (AAAAATTAAACGCTACTCCA) (Pais *et al*., 2008). For DNA quality control, the *G. morsitans* subsp. *morsitans tubulin* gene (accession no. DQ377071) were amplified with primers GmmTubF (TAGTTCTCTTCAACTTCAGCCTCTT) and GmmTubR (TCGTTGACCATGTCTGGTGT) (Pais *et al*., 2008). Bacteria-specific PCR amplification conditions consisted of initial denaturation at 94°C for 2 min, followed by 30 cycles of 94°C for 30 s, 54°C for 40 s and 72°C for 1 min with a final elongation at 72°C for 7 min. For *gmmtub* amplification, an annealing temperature of 60°C was used. The amplification products were analysed by agarose gel electrophoresis using ethidium bromide and visualized using a transilluminator (Pais *et al*., 2008).

ITS-PCR was undertaken in 25 *μ*L reaction mixtures containing primers AITS-F: CGGAAGTTCACCGATATTGC and AITS-R: AGGAAGCCAAGTCATCCATC (Gaithuma *et al*., 2019), One Taq 2 @ master mix (New England BioLabs, Ipswich, MA, USA), nuclease-free water and 5 *μ*L of extracted DNA sample. For the detection of *T. b. rhodesiense*, SRA F (5′-ATAGTGACAAGATGCGTACTCAACGC-3′) and SRA R (5′-AATGTGTTCGAGTACTTCGGTCACGCT-3′) (Radwanska *et al*., 2002) were used (procured from Inqaba Biotec, Pretoria, South Africa). Thermocycler amplification conditions were at 94°C for 5 min, followed by 40 cycles of 94°C for 40 s, 58°C for 40 s, 72°C for 90 min and 72°C for 5 min. ITS-PCR targets the internal transcribed spacer 1 of the ribosomal RNA (100–200 copies per genome), producing different sized products for different trypanosome species (Desquesnes *et al*., 2001; Njiru *et al*., 2005; Gaithuma *et al*., 2019). ITS-PCR products were separated by electrophoresis (95 V for 60 min) in a 2% (w/v) agarose gel containing ethidium bromide. The separated products were then visualized under ultraviolet light in a transilluminator. Known positive controls of *T. congolense*, *T. vivax*, *T. b. rhodesiense* and *T. brucei* and a negative control were included in each reaction. All samples that were positive for *T. brucei* were subjected to a multiple PCR using a serum resistance-associated antigen (SRA) targeting primer for the detection of *T. b. rhodesiense* (Welburn *et al*., 2001; Radwanska *et al*., 2002; Gaithuma *et al*., 2019).

### Statistical analysis

The prevalence data of trypanosome and symbiont infection from captured tsetse flies were summarized as frequencies and percentages and analysed using descriptive statistics in Epi-info 7.2. Odds ratios were used as measures of association. A chi-square test was used to determine statistical differences between proportions. For expected values under 5, Fisher's exact test was used. Statistical significance was acceptable at *P* < 0.05. Pearson's correlation test was used to see if the presence of symbionts correlated with the presence of trypanosomes. Scores were used to determine the degree of correlation present. The scale of correlation coefficients were classified as follows: negative values (negative association), positive values (positive association), no association (0.00), very low (0.00–0.19), low (0.20–0.39), moderate (0.40–0.69), high (0.70–0.89) and very high (0.90) (Schober *et al*., 2018).

## Results

The combined prevalence for *Sodalis* and *Wolbachia* in captured tsetse flies was 95.3% (*n* = 278, 95% CI = 92.8–97.8) while the overall trypanosome prevalence in captured tsetse flies was 25.5% (*n* = 278, 95% CI = 20.4–30.7). Trypanosome prevalence was 10.8% (*n* = 30, 95% CI = 7.1–14.4) for *T. brucei*, 1.4% (*n* = 4, 95% CI = 0.0–2.8) for both *T. congolense* and *T. vivax* and 0.7% (*n* = 2, 95% CI = −0.3–1.7) for *T. b. rhodesiense*.

Out of 278 tsetse flies that were captured for the study, a total of 237 (85.3%) flies belonged to the group of *Glossina pallidipes* while 41 (14.8%) were *G. morstitans morsitans*. Total symbiont infections in *G. pallidipes* was 94.9% (*n* = 225, 95% CI = 92.2–97.7) while in *G. m. morsitans* was 97.6% (*n* = 40, 95% CI = 92.8–102.3), trypanosome infections in *G. pallidipes* was 26.6% (*n* = 63, 95% CI = 21.0–32.2) while in *G. m. morsitans* was 19.5% (*n* = 8, 95% CI = 7.4–31.6). No significant difference was observed in both symbiont (*P* = 0.46) and trypanosome (*P* = 0.34) infections in the 2 tsetse species sampled. The prevalence of symbionts and trypanosomes in the 2 tsetse species detected by PCR is summarized in Table 1.

**Table 1.** Prevalence (%) of symbionts and trypanosomes in tsetse species captured in the Luangwa valley, eastern Zambia

Tsetse species	Symbionts		Trypanosomes					
	*Sodalis*	*Wolbachia*	*T. brucei*	*T. b. brucei*	*T. vivax*	*T. congolense*	*T. b. rhodesiense*	*Mixed infections*
*G. m. morsitans* Prevalence (95% CI)	85.4% (74.6–96.2)	80.5% (68.4–92.6)	19.5% (7.4–31.6)	19.5% (7.4–31.6)	0	0	0	0
*G. pallidipes* Prevalence (95% CI)	86.5% (82.2–90.9)	78.9% (73.7–84.1)	12.7% (8.4–16.9)	8.2% (4.6–11.5)	1.7% (0.1–3.3)	1.7% (0.1–3.3)	0.8% (−0.3–2.0)	1.7% (0.1–3.3)

The likelihood of female flies harbouring *Sodalis* (OR = 1.9, 95% CI 0.8–4.4) and *Wolbachia* (OR = 1.3, 95% CI 0.7–2.5) was higher than in male flies (Table 2).

**Table 2.** Symbiont and trypanosome infection in relation to the sex of caught tsetse flies in the Luangwa valley, eastern Zambia

	Symbionts		Trypanosomes			
	*Sodalis*	*Wolbachia*	*T. brucei*	*T. vivax*	*T. congolense*	*T. b. rhodesiense*
Female	158	146	17	2	1	1
Male	82	74	17	2	3	1
Odds ratio	1.9	1.3	2.3	2.1	6.4	2.1

Of the 240 tsetse flies that were positive for *Sodalis,* the prevalence of *T. brucei* was 12.9% (95% CI 8.7–17.2) while that of *T. congolense* was 1.7% (95% CI 0.1–3.3), *T. vivax* 1.3% (95% CI −0.2–2.7) and *T. b. rhodesiense* 0.8% (95% CI −0.3–2.0). Similarly, of the 220 tsetse flies that were positive for *Wolbachia*, trypanosome prevalence for *T. brucei* was 12.7% (95% CI 8.3–17.1) while that of *T. congolense* was 1.8% (95% CI 0.1–3.6), *T. vivax* 1.4% (95% CI −0.2–2.9) and *T. b. rhodesiense* 0.9% (95% CI −0.4–2.2).

Analysis of the association between trypanosomes and endosymbiont infection in the caught tsetse flies (Table 3) found a 1.3 (95% CI 0.5–3.2) times likelihood of *T. brucei* infection when *Wolbachia* is present and 1.7 (95% CI 0.5–6.0) likelihood of *T. brucei* infection when *Sodalis* is present. Similarly, results indicate a 0.8 (95% CI 0.1–7.7) likelihood of *T. vivax* infection when *Wolbachia* is present and a 0.5 (95% CI 0.0–4.6) likelihood of *T. congolense* infection when *Sodalis* is present.

**Table 3.** Measures of association between trypanosome and symbiont infection in tsetse flies caught in the Luangwa valley, eastern Zambia

		*Wolbachia* infection		Odds ratio	Confidence interval at 95%	*Sodalis* infection		Odds ratio	Confidence interval at 95%
	*Trypanosome* infection	Present	Absent			Present	Absent		
*T. brucei*	Present	192	52	1.3	0.5–3.2	209	35	1.7	0.5–6.0
	Absent	28	6			31	3		
*T. congolense*	Present	216	58	–	–	237	37	0.5	0.0–4.6
	Absent	4	0			3	1		
*T. vivax*	Present	217	57	0.8	0.1–7.7	236	38	–	–
	Absent	3	1			4	0		
*T. b. rhodesiense*	Present	218	58	–	–	238	38	–	–
	Absent	2	0			2	0		

Analysis of the correlation between the presence of tsetse endosymbionts and trypanosome infection showed no correlation (Table 4).

**Table 4.** Correlations between trypanosome and symbiont infection in tsetse flies caught in the Luangwa valley, eastern Zambia

	*T. brucei*		*T. vivax*		*T. congolense*		*T. b. rhodesiense*	
	Pearson's correlation	Sig. (2-tailed)	Pearson's correlation	Sig. (2-tailed)	Pearson's correlation	Sig. (2-tailed)	Pearson's correlation	Sig. (2-tailed)
*Sodalis*	0.05	0.38	0.05	0.43	−0.04	0.51	0.03	0.57
*Wolbachia*	0.03	0.62	−0.01	0.84	0.06	0.30	0.04	0.47

## Discussion

The tsetse fly has established symbiotic associations with bacteria which influence its reproduction, nutrition and vector competence. Symbiotic interactions are widespread in insects (and also animals and plants) and may provide an avenue for disease control (Ricci *et al*., 2012; Wamiri *et al*., 2013). The current study provided the prevalence of selected tsetse symbionts and trypanosomes in *Glossina* tsetse species from eastern Zambia. Results showed no statistical difference in the prevalence of both symbionts and trypanosomes in the 2 tsetse species (*G. m. morsitans* and *G. pallidipes*) analysed. No association was either observed between symbiont and trypanosome infection in the 2 tsetse species, suggesting that endosymbionts play no role in tsetse vector competence and reproduction in the area. These data are in agreement with those obtained by Dennis *et al*. (2014) but disagree with those by Farikou *et al*. (2010) and Mbewe *et al*. (2015), who established the existence of a relationship between tsetse bacteria and trypanosomes and the potential role of endosymbionts in tsetse vector competence and reproduction. However, later studies were conducted in different geographical areas with different species of tsetse flies (*G. p. palpalis* and *G. m. centralis*, respectively).

Tsetse symbionts (*Wolbachia* and *Sodalis*) were detected in about 95% of the tsetse samples examined with varying prevalence within tsetse species. Both symbionts were found in relative abundance in the 2 tsetse species examined, with *Sodalis* prevalence slightly higher than *Wolbachia*. This agrees with findings from similar studies on tsetse symbionts though with varying levels of infection rates which may be attributed to differences in the sensitivity of the screening methods (Doudoumis *et al*., 2012; Dennis *et al*., 2014; Doudoumis *et al*., 2017). The low numbers of *Wolbachia* have been associated with low sensitivity of the standard PCR assay (Wamiri *et al*., 2013), which was also used in our laboratory analysis of tsetse samples. The presence of *Sodalis* and *Wolbachia* infection in the tsetse population sampled re-affirms the presence of tsetse bacterium in tsetse species found in Zambia and particularly the Luangwa valley (Doudoumis *et al*., 2012; Dennis *et al*., 2014; Mbewe *et al*., 2015).

The overall trypanosome prevalence in the captured tsetse flies (25.5%) was similar to what was found by Nakamura *et al*. (2021). The identification of *T. congolense, T. brucei* and *T. vivax* from tsetse samples analysed confirms the presence of AAT in the community (Mekata *et al*., 2008; Laohasinnarong *et al*., 2015; Mulenga *et al*., 2021; Nakamura *et al*., 2021). The presence of *T. b. rhodesiense* further indicated the circulation of the human-infective trypanosomes in the area, responsible for sleeping sickness and the importance of the tsetse species in trypanosomiasis transmission. Taken together, the presence of pathogenic trypanosomes in tsetse species examined provide insights to the risk of contracting sleeping sickness and AAT by the local communities and their livestock (Mekata *et al*., 2008; Djohan *et al*., 2015; Auty *et al*., 2016).

In agreement with Mekata *et al*. (2008), high infections of both symbionts and trypanosomes were reported in the *G. pallidipes* species compared to *G. m. morsitans*. However, unlike observations from the current study, Doudoumis *et al*. (2012) found *G. m. morsitans* to be more likely to harbour *Wolbachia* than *G. pallidipes*. On the other hand, current study findings were in concordance with findings obtained elsewhere, where *G. pallidipes* was captured with other tsetse species other than *G. morsitans* (Wamiri *et al*., 2013). Further, the high prevalence of female *G. pallidipes* found agree with findings by Laohasinnarong *et al*. (2015). Overall, both symbiont and trypanosome prevalence were, however, higher in female tsetse flies than in male tsetse flies and were associated with the host tsetse species as previously reported (Wamiri *et al*., 2013; Dennis *et al*., 2014). Such findings prompt for further research in the importance of *G. pallidipes* tsetse species with regards to host genetic diversity and vectoral capacity in areas where other tsetse species are present.

The weak relationship between tsetse symbiont prevalence and trypanosome prevalence shown in the current study does not support the synergistic role between symbiont and trypanosomiasis transmission in the surveyed area. However, the low number of tsetse flies infected with trypanosomes could explain the poor correlation observed, which suggest the need for further work on the importance of *Sodalis* in tsetse species in the Luangwa valley tsetse belt. Understanding insect–parasite–symbiont interactions is necessary in establishing opportunities for biologically based trypanosomiasis control strategies (Boulanger *et al*., 2002). The importance of understanding this relationship is emphasized by the urgent need for environmentally friendly methods for both tsetse and trypanosomiasis control. The high prevalence of *Wolbachia* in female flies need to be investigated further as a possible basis for environmentally sustainable tsetse population control for *Glossina* species.

## Data Availability

The data that support the findings of this study are available from the corresponding author upon reasonable request.
